# Validation of Noninvasive Assessment of Pulmonary Gas Exchange in Patients with Chronic Obstructive Pulmonary Disease during Initial Exposure to High Altitude

**DOI:** 10.3390/jcm12030795

**Published:** 2023-01-19

**Authors:** Benoit Champigneulle, Lukas Reinhard, Maamed Mademilov, Mathieu Marillier, Tanja Ulrich, Arcangelo F. Carta, Philipp Scheiwiller, Saltanat B. Shabykeeva, Ulan U. Sheraliev, Ainura K. Abdraeva, Kamila M. Magdieva, Gulzada Mirzalieva, Aijan T. Taalaibekova, Aigul K. Ozonova, Aidai O. Erkinbaeva, Nurdin U. Shakiev, Syimyk A. Azizbekov, Philip N. Ainslie, Talant M. Sooronbaev, Silvia Ulrich, Konrad E. Bloch, Samuel Verges, Michael Furian

**Affiliations:** 1 HP2 Laboratory, INSERM U1300, Grenoble Alpes University, CHU Grenoble Alpes, 38400 Grenoble, France; 2 Department of Anesthesia and Critical Care, Grenoble Alpes University Hospital, 38043 Grenoble, France; 3 Department of Pulmonology, University Hospital Zürich, 8091 Zurich, Switzerland; 4 Swiss-Kyrgyz High-Altitude Medicine and Research Initiative, 8091 Zurich, Switzerland; 5 Swiss-Kyrgyz High-Altitude Medicine and Research Initiative, Bishkek 720040, Kyrgyzstan; 6 Department of Respiratory Medicine, National Center for Cardiology and Internal Medicine, Bishkek 720040, Kyrgyzstan; 7 Centre for Heart, Lung and Vascular Health, School of Health and Exercise Sciences, University of British Columbia Okanagan, Kelowna, BC V1V 1V7, Canada

**Keywords:** gas exchange, O_2_ deficit, noninvasive measurement, COPD, validation, AGM100

## Abstract

Investigation of pulmonary gas exchange efficacy usually requires arterial blood gas analysis (aBGA) to determine arterial partial pressure of oxygen (mPaO_2_) and compute the Riley alveolar-to-arterial oxygen difference (A-aDO_2_); that is a demanding and invasive procedure. A noninvasive approach (AGM100), allowing the calculation of PaO_2_ (cPaO_2_) derived from pulse oximetry (SpO_2_), has been developed, but this has not been validated in a large cohort of chronic obstructive pulmonary disease (COPD) patients. Our aim was to conduct a validation study of the AG100 in hypoxemic moderate-to-severe COPD. Concurrent measurements of cPaO_2_ (AGM100) and mPaO_2_ (EPOC, portable aBGA device) were performed in 131 moderate-to-severe COPD patients (mean ±SD FEV_1_: 60 ± 10% of predicted value) and low-altitude residents, becoming hypoxemic (i.e., SpO_2_ < 94%) during a short stay at 3100 m (Too-Ashu, Kyrgyzstan). Agreements between cPaO_2_ (AGM100) and mPaO_2_ (EPOC) and between the O_2_-deficit (calculated as the difference between end-tidal pressure of O_2_ and cPaO_2_ by the AGM100) and Riley A-aDO_2_ were assessed. Mean bias (±SD) between cPaO_2_ and mPaO_2_ was 2.0 ± 4.6 mmHg (95% Confidence Interval (CI): 1.2 to 2.8 mmHg) with 95% limits of agreement (LoA): −7.1 to 11.1 mmHg. In multivariable analysis, larger body mass index (*p* = 0.046), an increase in SpO_2_ (*p* < 0.001), and an increase in PaCO_2_-PETCO_2_ difference (*p* < 0.001) were associated with imprecision (i.e., the discrepancy between cPaO_2_ and mPaO_2_). The positive predictive value of cPaO_2_ to detect severe hypoxemia (i.e., PaO_2_ ≤ 55 mmHg) was 0.94 (95% CI: 0.87 to 0.98) with a positive likelihood ratio of 3.77 (95% CI: 1.71 to 8.33). The mean bias between O_2_-deficit and A-aDO_2_ was 6.2 ± 5.5 mmHg (95% CI: 5.3 to 7.2 mmHg; 95%LoA: −4.5 to 17.0 mmHg). AGM100 provided an accurate estimate of PaO_2_ in hypoxemic patients with COPD, but the precision for individual values was modest. This device is promising for noninvasive assessment of pulmonary gas exchange efficacy in COPD patients.

## 1. Introduction

In clinical practice, sampling arterial blood to measure the partial pressures of oxygen (PaO_2_) and carbon dioxide (PaCO_2_) remains a keystone to investigating pulmonary gas exchange abnormalities [1]. Additionally, using the Riley ideal alveolar partial pressure of oxygen (PAO_2_) equation [2], the traditional alveolar-to-arterial oxygen difference (A-aDO_2_) can be calculated [3]. This approach is, for instance, helpful to assess and follow up over time the decline of gas exchange accountable for diffusion or ventilation-to-perfusion ratio ($\dot{V_{A}}/\dot{Q}$) impairments in patients with chronic lung diseases [4,5]. Furthermore, for these patients, the PaO_2_ value may lead to therapeutic decisions such as long-term oxygen therapy [6]. However, this traditional assessment strategy has some disadvantages: arterial puncture often remains a painful experience for patients, and puncture failure is not unusual [7]. Arterial blood gas analysis (aBGA) also requires expensive analyzers and trained operators, which is not compatible with an easy pulmonary gas exchange assessment outside hospital facilities. Thus, well-validated, reproductible, and easy-to-perform gas exchange assessment options are currently lacking [8].

Recently, a non-invasive approach for measuring the pulmonary gas exchange has been developed and commercialized (AGM100^TM^, MediPines Corp., Yorba Linda, CA, USA). AGM100 requires sampling partial pressure of end-tidal oxygen (PETO_2_) and carbon dioxide (PETCO_2_) during quiet and steady breathing (thus reflecting intra-alveolar values); the PaO_2_ is calculated (cPaO_2_) from oxygen saturation measured through a pulse oximeter (SpO_2_) using the oxygen–hemoglobin dissociation curve with a PaCO_2_-shift-correction based on PETCO_2_ values [9,10]. This approach allows the computation of the O_2_ deficit, defined as the difference between alveolar PO_2_ and cPaO_2_, which has been suggested to be a reliable surrogate of the conventional A-aDO_2_ and $\dot{V_{A}}/\dot{Q}$ mismatch [11]. This O_2_ deficit has been shown indeed to strongly correlate with the conventional A-aDO_2_ in hypoxemic patients [12] and in healthy volunteers exercising in a hypoxic environment [13]. Moreover, the O_2_ deficit is elevated with normal ageing and augmented in patients with lung diseases when compared to healthy young volunteers [14,15]. Furthermore, the cPaO_2_ has been shown to be a valid estimation of the measured PaO_2_ (mPaO_2_) in healthy, hypoxic subjects achieving a progressive cycling test in normobaric hypoxia [13].

This new and non-invasive pulmonary gas exchange measurement may thus represent a promising method for the assessment of gas exchange impairment [8,11]. One small validation study (*n* = 23) reported a mean bias of −4 mmHg between cPaO_2_ and mPaO_2_ in a heterogeneous group of patients [12]. However, validation data and insights into the accuracy of such non-invasive measurements in particular are currently lacking in a large and homogenous population of patients suffering from chronic lung diseases, such as chronic obstructive pulmonary disease (COPD). COPD is a highly prevalent disease and represents the third leading cause of death worldwide [16,17]. In these patients, disease progression gradually leads to hypoxemia, mainly through $\dot{V_{A}}/\dot{Q}$ impairments [18], which requires regular follow-up of pulmonary gas exchange efficiency, including aBGA [4,5,16].

Therefore, the aim of this diagnostic accuracy study was to compare the cPaO_2_ and O_2_ deficit obtained from the AGM100 to PaO_2_ and A-aDO_2_ obtained and calculated from aBGA in a homogenous population of COPD patients becoming hypoxemic during a short high-altitude sojourn.

## 2. Methods

### 2.1. Study Design and Participants

This study was conducted within a large research project conducted in Kyrgyzstan in 2021, which involved stable, moderate-to-severe COPD patients exposed to a high-altitude environment (High Altitude Clinic, Too-Ashu, 3100 m) during a 2-day period (ClinicalTrials.gov NCT03957759 and NCT04913389). The study was approved by the Ethics Committee of the National Center for Cardiology and Internal Medicine (01-2021, Bishkek, Kyrgyzstan) and was conducted in accordance with the Declaration of Helsinki. All participants were fully informed in their native language and provided a written consent.

Participants included were patients aged between 35 and 75 years, with stable, moderate-to-severe COPD diagnosed according to the Global initiative for Obstructive Lung Disease (GOLD) guidelines [16] with a forced expiratory volume in the first second of expiration (FEV_1_) between 40 and 80% of predicted value and a resting SpO_2_ < 94% at 3100 m. All patients were living at low altitude (<1000 m) and free of other unstable comorbidities.

### 2.2. Experimental Protocol

All measurements were conducted in a supine, bedrest position with the head at 10–20°. No position change was allowed between the AGM100 measurement and the arterial puncture. Measurements were conducted at different time points while patients were exposed to a hypobaric environment at 3100 m: (1) at 6:00 AM after the first night at 3100 m while patients were awake but still in bed or (2) in patients who prematurely, i.e., before the first night at high altitude, experienced an altitude-related adverse health effect (ARAHE, a composite criterion including a severe hypoxemia defined as resting SpO_2_ < 80% over 30 min or a resting SpO_2_ < 75% over 15 min) [19]. In case of an ARAHE, AGM100 measurement and aBGA were performed before starting oxygen therapy or after stopping oxygen therapy for at least 20 min. Procedures were standardized as follows:

- Non-invasive AGM100 measurement was first performed: participants were asked to breathe through a mouthpiece (with a nose clip) to record PETO_2_ and PETCO_2_, while SpO_2_ was continuously measured with a finger pulse oximeter, connected to the device. After automatic detection of a breathing steady-state, the measurement was automatically stopped and values for SpO_2_, PETO_2_, and PETCO_2_ were recorded, and the cPaO_2_ and O_2_ deficit were calculated [10,11].
- Immediately after the AGM100 measurement, an arterial blood sample was collected by radial artery puncture while participants were breathing ambient air. Each sample was analyzed using a point-of-care blood gas analyzer (EPOC^®^, Siemens Healthcare, Erlangen, Germany). PaO_2_, PaCO_2_, and arterial pH were analyzed. The EPOC has been previously validated in a high-altitude environment [20].

Using the mPaO_2_ from the EPOC, the conventional A-aDO_2_ was calculated [1]. Calculated PAO_2_ (cPAO_2_) was obtained using the alveolar gas equation [3]: ${\mathrm{cPAO}}_{2}={\mathrm{FiO}}_{2}\times (P_{\mathrm{Atm}}-P_{H2O})-\frac{{\mathrm{PaCO}}_{2}}{\mathrm{RER}}\times \left[1-{\mathrm{FiO}}_{2}\times \left(1-\mathrm{RER}\right)\right]$ where the respiratory exchange ratio (RER) was assumed to be equal to 0.8; P_Atm_ represents the atmospheric pressure; and P_H2O_ represents the saturated water vapor pressure at 37 °C (47 mmHg).

### 2.3. Clinical Assessment

For each patient, the medical history was obtained, and a clinical examination was performed prior to the inclusion in the main study at low altitude (Bishkek, Kyrgyzstan, 760 m). At the same time, spirometry was performed to confirm the airflow obstruction and COPD severity according to standard guidelines [21]. Severity of COPD was classified according to the GOLD grade [16]. Assessment of breathlessness and life-impact of COPD were evaluated using the modified British medical research council (mMRC) and the COPD assessment test (CAT) scores [16].

### 2.4. Outcomes

The main outcome of this validation study was the accuracy and precision of cPaO_2_ (AGM100) in comparison to mPaO_2_ (EPOC). Secondary outcomes included the identification of factors associated with the imprecision of the cPaO_2_ estimation, the diagnostic performance of the AGM100 to detect a predefined severe resting hypoxemia, and the agreement between the O_2_ deficit (AGM100) and the A-aDO_2_ (EPOC).

### 2.5. Statistical Analysis

Data reporting: continuous variables are presented as mean ± standard deviation (SD) or median (25–75th percentiles) as appropriate. Categorical variables were reported in numbers and percentages (%).

Agreement analysis between AGM100 and EPOC: agreement between cPaO_2_ (AGM100) and mPaO_2_ (EPOC), between PETCO_2_ (AGM100) and mPaCO_2_ (EPOC), and between O_2_ deficit (AGM100) and A-aDO_2_ (EPOC) were assessed using linear regression analyses, computation of Pearson correlation coefficients, and Bland–Altman plotting [22]. As an estimate of accuracy, mean bias ± SD with 95% confidence interval (CI) was calculated; as an estimate of precision, the upper and lower limits of agreements (LOA) were computed [23]. The possibility of a proportional bias was evaluated using a linear regression analysis on the Bland–Altman plot [23]. Bland–Altman was plotted considering the absolute difference against the mean value for PaO_2_, whereas absolute difference between O_2_ deficit and A-aDO_2_ was plotted against A-aDO_2_, considering A-aDO_2_ as the “gold standard” [24]. Furthermore, as PETCO_2_ is used as a surrogate of PaCO_2_ to correct the cPaO_2_ from SpO_2_ [9,10], agreement between the two values was also assessed using both linear regression and Bland–Altman plotting.

Sensitivity analysis: since aBGA point-of-care devices as EPOC may not be considered as accurate as stationary devices, we conducted a sensitivity analysis for PaO_2_ and A-aDO_2_ agreements, using a corrected value of mPaO_2_ computed from the regression equation developed in a previous validation study of the EPOC, conducted in similar field conditions [20].

Multivariable regression analysis: to assess confounding factors that may explain a discrepancy between cPaO_2_ and mPaO_2_, we performed a multivariable regression analysis, considering the difference between the two values as the dependent variable and age, sex, body mass index, SpO_2_, and time delay between the two measurements and PaCO_2_-PETCO_2_ gradient as potential explanatory variables. A parsimonious model was built using a backward stepwise elimination of the most non-significant variables; therefore, the only significant explanatory variables were retained in the final model.

Diagnosis performance: the predictive performance of the AGM100 to detect predefined severe resting hypoxemia of PaO_2_ < 60 mmHg and PaO_2_ ≤ 55 mmHg (versus EPOC mPaO_2_) was investigated by computation of the sensitivity (Sn), specificity (Sp), positive predictive value (PPV), negative predictive value (NPV), and positive likelihood ratio (LR+). Both hypoxemia thresholds are commonly admitted for home oxygen therapy in COPD [6].

All tests were two-sided, and a *p*-value < 0.05 or a 95% CI excluding zero was considered statistically significant. All statistical analyses were performed using R software (version 4.1.2, The R Foundation for Statistical Computing, Vienna, Austria) and GraphPad Prism (version 9.3.1, GraphPad Software, Boston, MA, USA).

## 3. Results

### 3.1. Measurements and Patients Included in the Study

During the study period, 153 single AGM100 measurements were performed in 153 COPD patients sojourning at 3100 m. Among them, 12 were not paired with an aBGA due to arterial puncture failures (*n* = 12, 8% failure rate), and 10 measurements were excluded from the analysis due to a SpO_2_ ≥ 94% at the time of measurement. Thus, 131 paired AGM100-EPOC measurements obtained from 131 COPD patients were included in the analysis. Among them, 110 measurements (84%) were preplanned measurements (i.e., conducted at 06:00 AM after the first night at 3100 m), whereas 21 measurements (16%) were non-planned measurements performed when experiencing an ARAHE (mostly isolated severe hypoxemia). A steady-state was automatically reached in 65 (60–81) seconds, for all, except one, measurements. Median delay between the end of the AGM100 measurement and the arterial puncture was 217 (153–384) seconds, and aBGA was performed at 163 (109–285) seconds after the arterial puncture. Mean SpO_2_ at the time of the AGM100 measurement was 87 ± 4% (range: 74 to 93%). Demographic characteristics of the patients are presented in the Table 1.

### 3.2. PaO_2_ Agreement between cPaO_2_ (AGM100) and mPaO_2_ (EPOC)

**Figure 1:**
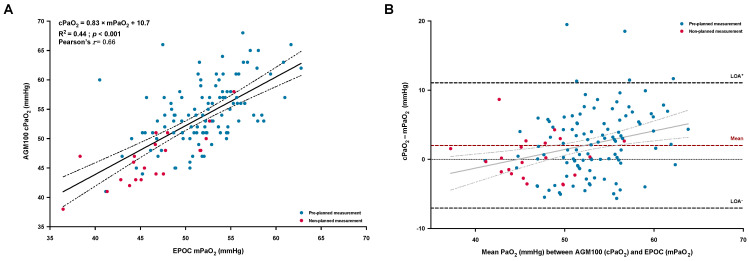
Linear regression and correlation (**A**) between the measured arterial PaO_2_ (mPaO_2_) with the portable blood gas analyzer (EPOC) and the calculated PaO_2_ (cPaO_2_) provided by the AGM100 device; the solid and dashed lines represent the regression line with the 95% confident interval limits. Bland–Altman plot of agreement (**B**) between the cPaO_2_ and the mPaO_2_, expressed as absolute differences vs. the mean of both measurements; the dashed lines represent the mean bias (in red) and the 95% limits of agreement (LOA, in black). Individual values were identified as pre-planned measurement (early morning after a first night at high altitude, blue solid circles) or as non-planned measurement (occurrence of an early altitude-related adverse health effect, red solid circles).

A moderate but significant correlation was observed between cPaO_2_ and mPaO_2_ (Figure 1A). Furthermore, the two methods showed good accuracy with a low mean positive bias, albeit significant (Figure 1B and Table 2), but a low precision according to the large variability (Figure 1B and Table 2). Linear regression analysis (Figure 1B) highlighted a proportional bias, which increased with higher values of PaO_2_ (*p* = 0.004). Sensitivity analysis using the corrected mPaO_2_ (Table 2) did not show any improvement in agreement between the two values, with a low mean negative bias.

### 3.3. Agreement between PETCO_2_ (AGM100) and PaCO_2_ (EPOC)

**Figure 2:**
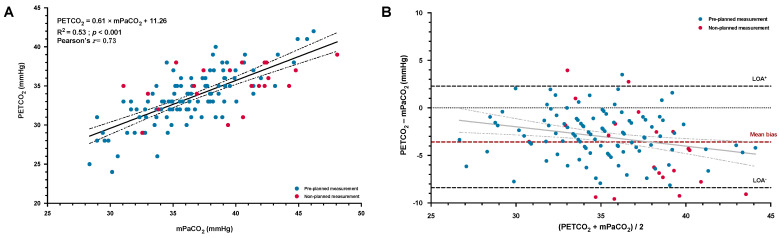
Linear regression and correlation (**A**) between the measured arterial PaCO_2_ (mPaCO_2_) with the portable blood gas analyzer (EPOC) and the end-tidal CO_2_ (PETCO_2_) obtained from the AGM100; the solid and dashed lines represent the regression line with the 95% confident interval limits. Bland–Altman plot of agreement (**B**) between the PETCO_2_ and the mPaCO_2_, plotted as absolute differences vs. the mean of both measurements; the dashed lines represent the mean bias (in red) and the 95% limits of agreement (LOA, in black). Individual values were identified as pre-planned measurement (early morning after a first night at high altitude, blue solid circles) or as non-planned measurement (occurrence of an early altitude-related adverse health effect, red solid circles).

A moderate and significant correlation was shown between the mPaCO_2_ and the PETCO_2_ (Figure 2A). Bland–Altman analysis (Figure 2B) among the two variables highlighted a low and significant mean bias of −3.1 ± 2.7 mmHg (95% CI, −3.5 to −2.6 mmHg), which increased with higher values of PaCO_2_ (*p* = 0.004).

### 3.4. Factors Associated with the Accuracy of the cPaO_2_ and Diagnosis Performance

Multivariable linear analysis including both demographic and procedural factors (Table 3) highlighted that the increase in body mass index and in PaCO_2_-PETCO_2_ gradient were positively associated with an increase in discrepancy between cPaO_2_ and mPaO_2_. Conversely, lower SpO_2_ were independently associated with a better accuracy (i.e., a lower absolute difference) between cPaO_2_ and mPaO_2_. When considering the predictive diagnosis performance (Table 4), the AGM100 device showed a high PPV to detect a severe resting hypoxemia for both considered thresholds of PaO_2_.

### 3.5. Agreement between O_2_ Deficit and A-aDO_2_

**Figure 3:**
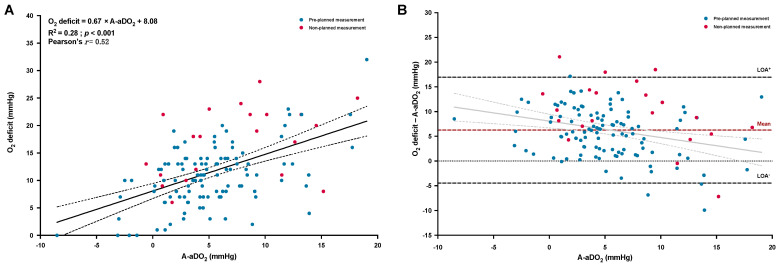
Linear regression and correlation (**A**) between the traditional alveolar-to-arterial oxygen difference (A-aDO_2_) and the oxygen deficit (O_2_ deficit); the solid and dashed lines represent the regression line with the 95% confident interval limits. Bland–Altman plot of agreement (**B**) between the O_2_ deficit and the A-aDO_2_, plotted as the absolute difference vs. A-aDO_2_; the dashed lines represent the mean bias (in red) and the 95% limits of agreement (LOA, in black). Individual values were identified as pre-planned measurement (early morning after a first night at high altitude, blue solid circles) or as non-planned measurement (occurrence of an early altitude-related adverse health effect, red solid circles).

Patients exhibited larger values for O_2_ deficit than for A-aDO_2_ (11.8 ± 6.1 vs. 5.5 ± 4.8 mmHg, respectively, *p* < 0.001) that seemed mostly explained by the difference between measured PETO_2_ and cPAO_2_ (mean difference: 8.2 ± 4.4 mmHg, 95% CI, 7.4 to 8.9 mmHg), rather than by the difference between cPaO_2_ and mPaO_2_ (mean difference: 2.0 ± 4.6 mmHg, 95% CI, 1.2 to 2.8 mmHg). O_2_ deficit showed a moderate but significant correlation with A-aDO_2_ (Figure 3A) with a global positive and significant mean bias of 6.2 ± 5.5 mmHg (Table 2 and Figure 3B). Neither O_2_ deficit nor A-aDO_2_ were significantly correlated with pulmonary function (FEV_1_ and FVC, all *p* > 0.05). Sensitivity analysis using the corrected values of mPaO_2_ to compute A-aDO_2_ (Table 2) led to a larger bias among the two parameters.

## 4. Discussion

Until now, reports of clinical application of the AGM100 remained anecdotal and confined to patients with acute respiratory failure [25,26], mainly due to the novelty of this method. To the best of our knowledge, this research represents the widest validation study of such non-invasive gas exchange assessment method in a homogenous cohort of hypoxemic patients with moderate-to-severe COPD, following a short high-altitude exposure. The main findings indicate the ability of the AGM100 to predict PaO_2_ (with an absolute low mean bias of 2 mmHg versus measured PaO_2_ with a previously validated point-of-care analyzer [20]) and the satisfying diagnosis predictive performance (Table 4) of this device to diagnose severe resting hypoxemia. Moreover, the AGM100 provides an estimation of the pulmonary gas exchange efficacy through the O_2_ deficit. Taken together, these results suggest that this non-invasive method may serve as a tool to investigate gas exchanges in hypoxemic COPD patients. Such option may be particularly relevant when aBGA are not available or fail.

Despite our promising results, some aspects of the agreement between methods need to be discussed. Even though we reported a similar positive mean bias (2.0 ± 4.6 mmHg) to the one previously reported in a small and heterogenous sample of 23 hypoxemic patients (2.7 ± 7.0 mmHg) [12], the accuracy reported by Howe et al. [13] in healthy volunteers via an intra-arterial catheter during rest and exercise in hypoxia was slightly better (mean bias of 1.0 ± 2.8 mmHg). Moreover, the precision of the cPaO_2_ could probably be improved, as suggested by our results, regarding the high observed dispersion around the mean bias, albeit concordant with previous results [12]. In particular, we identified that the inaccuracy (i.e., the increase in cPaO_2_-mPaO_2_ difference) of the measurement was independently associated with the difference between PaCO_2_ and PETCO_2_. This statistical relationship may partially explain some errors in the estimated PaO_2_, as the algorithm of the AGM100, considering the Bohr effect, corrects the cPaO_2_ using the PETCO_2_ [10,11]. Measurement of PETCO_2_ in healthy people breathing a hypoxic mixture has been shown to be highly reproducible and can be considered as a reliable surrogate of PaCO_2_ [27]. However, the reliability of this estimation may be lower in patients with $\dot{V_{A}}/\dot{Q}$ abnormalities such as COPD [28], being even more pronounced at altitude (as reflected by an increase in dead space fraction) [29]. The previously suggested relative inability of the AGM100 to compute an accurate cPaO_2_ in a non-hypoxemic patient (due to the flat portion of the hemoglobin dissociation curve) [9,14,15] was evidenced in our study by the proportional bias (Figure 1B) and by the independent association between greater SpO_2_ and larger divergence between cPaO_2_ and mPaO_2_. However, as previously emphasized, this technical limitation is, by itself, not a major problem because gas exchange impairments are unlikely in non-hypoxemic subjects [27]. Therefore, according to previous studies [12,14,15], we did not include non-hypoxemic COPD patients (SpO_2_ ≥ 94%) at the time of the pre-planned aBGA.

The O_2_ deficit, promptly computed by the AGM100 from the measured PETO_2_ and cPaO_2_ may represent another important clinical parameter similar to A-aDO_2_ to investigate the underlying mechanisms of hypoxemia [11]. Such alternative may also be a paradigm shift compared to the A-aDO_2_, since PaO_2_ and PAO_2_ are inversely calculated or measured among the two methods [9,11]. This allows the O_2_ deficit option to better considers lung units with high $\dot{V_{A}}/\dot{Q}$ ratios compared to the A-aDO_2_ [9,11,27]. This may be of particular interest in chronic lung diseases with $\dot{V_{A}}/\dot{Q}$ mismatches, especially in patients with COPD and emphysematous phenotype that typically leads to pulmonary areas with high $\dot{V_{A}}/\dot{Q}$ ratio [18,30]. Thus, as expected and previously reported, we observed larger values for O_2_ deficit than for A-aDO_2_ [12,13]. Furthermore, COPD patients included in our study exhibited higher O_2_ deficit than healthy young subjects breathing a 12.5% O_2_ mixture (corresponding to an elevation of ~3800 m) [15] and older people in similar conditions [31] but remained lower than measured in hospitalized patients [12]. The positive difference between O_2_ deficit and A-aDO_2_ observed in our study was mainly explained by a larger discrepancy between PETO_2_ and cPAO_2_ rather than between cPaO_2_ and mPaO_2_. This observation indicates that, in our cohort of COPD patients, the ideal cPAO_2_ (that did not consider the contribution of the lung units with high $\dot{V_{A}}/\dot{Q}$ ratio) underestimated the true mixed value of alveolar PO_2_. Other methodological consideration may include that the alveolar gas equation assumed a resting RER value of 0.8 [3], which was probably underestimated at high altitude, leading to underestimating the “true” PAO_2_ value. Thus, this pitfall may also explain the aberrant negative A-aDO_2_ values observed (Figure 3A), as also noted in the study conducted by Howe et al. in healthy people [13]. Nevertheless, a significant (but moderate) relationship was still observed between O_2_ deficit and A-aDO_2_ (Figure 3A) as previously shown [12,13]. We acknowledge, however, that this relationship and exploration of the agreement of O_2_ deficit and A-aDO_2_ is somewhat limited due to the inclusion of negative values from the A-aDO_2_ measures (that are due to technical rather than physiological differences), and PaO_2_ and PAO_2_ are inversely calculated or measured among the two methods; therefore, unlike PaO_2_, there is no real gold-standard comparison.

Our study has some methodological limitations. One may argue that the two measurements were not conducted simultaneously, and then ventilation and gas exchange may have been altered during this timeframe. However, similar to a previous study [12], this delay was reduced to only a few minutes with no position change to avoid postural gas exchange modifications [32]. Otherwise, we did not exactly assess the skin pigmentation of the participants; that may be an inaccuracy factor in SpO_2_ measurement (and so in PaO_2_ computation by the AGM100) for the darkest skin tones [33]. However, patients included in the present study, all natives from Kyrgyzstan in Central Asia, had skin tones mostly ranging from fair to moderate-brown skin (type II to IV on the Fitzpatrick scale) that probably did not induce significant SpO_2_ misestimations.

Another potential limitation for the transposition of our findings to clinical settings (i.e., the use of the AGM100 for long-term follow-up or acute assessment during COPD exacerbation) is that the primary mechanism of hypoxemia in our study is mainly driven by a decrease in PAO_2_ due to the hypobaric environment without any other cause of worsening hypoxemia (mainly involved in acute or chronic decrease in gas exchange efficiency in COPD). However, impairment of gas exchange in patients with COPD exposed to high altitude may also involve diffusion limitation and worsening of pulmonary hypertension [34,35].

## 5. Conclusions

The AGM100 proved a reliable and promising non-invasive method of gas exchange assessment in a large population of hypoxemic patients with moderate-to-severe COPD exposed to high altitude. These findings may be of strong interest for long-term follow-up, repetitive, or acute assessments of pulmonary gas exchange, especially in places where medical resources are limited. Further studies are required to specify the potential usefulness and added clinical value of the non-invasive gas exchange assessment in COPD patients or other chronic respiratory diseases. Especially, the ability of the AGM100 to estimate gas exchange changes over the time versus changes measured by aBGA remains to be investigated.

## Figures and Tables

**Table 1:** Participant characteristics.

	COPD Participants (*n* = 131)
Sex	
Men	70 (53%)
Women	61 (47%)
Age (years)	60 (53–65)
Body mass index (kg·m^−2^)	27.9 ± 4.0
Baseline SpO_2_ (%) at 760 m	95 ± 2
FEV_1_ (% predicted value)	60 ± 10
GOLD grade ^a^	
2	112 (85%)
3	19 (15%)
Smoking status ^b^	
Active smoker	20 (16%)
Ex-smoker	42 (34%)
Never smoke	62 (50%)
Smoking, pack-years	18 (8–40)
mMRC dyspnea score	1 (1–2)
CAT score	5 (3–9)
Comorbidities	
Hypertension	20 (15%)
Coronary artery disease	0 (0%)
Diabetes	4 (3%)
Others	13 (10%)
Pulmonary medication	
Inhaled beta-adrenergics	25 (19%)
Inhaled anticholinergics	51 (39%)
Inhaled corticosteroids	24 (18%)

Data are reported in mean ± SD, median (25–75th percentiles) or number (%) as appropriate. COPD, chronic obstructive pulmonary disease; SpO_2_, oxygen saturation assessed by finger oximetry; FEV_1_, forced expiratory volume in the first second of expiration; GOLD, Global initiative for chronic obstructive lung disease; mMRC, modified British medical research council; CAT, COPD assessment test. ^a^ GOLD grade 2, moderate COPD: postbronchodilator FEV_1_/FVC < 0.7, FEV_1_: 50 to 79% predicted value; GOLD grade 3, severe COPD: FEV_1_/FVC < 0.7, FEV_1_: 30 to 49% predicted value. ^b^ Data are missing for seven patients.

**Table 2:** Agreement among parameters derived from the AGM100 and EPOC devices.

Compared Variables	Mean Bias ± SD (mmHg)	95% CI Mean Bias (mmHg)	LOA (mmHg)
cPaO_2_ vs. mPaO_2_	2.0 ± 4.6	1.2 to 2.8	−7.1 to 11.1
cPaO_2_ vs. mPaO_2_ corrected ^a^	−2.3 ± 4.6	−3.1 to −1.5	−11.3 to 6.6
O_2_ deficit vs. A-aDO_2_	6.2 ± 5.5	5.3 to 7.2	−4.5 to 17.0
O_2_ deficit vs. A-aDO_2_ corrected ^a^	10.6 ± 5.5	9.6 to 11.5	−0.2 to 21.4

cPaO_2_, calculated arterial oxygen partial pressure (AGM100); mPaO_2_, measured arterial oxygen partial pressure (EPOC); SpO_2_, pulse oxygen saturation; O_2_ deficit, oxygen deficit (AGM100); A-aDO_2_, alveolar-to-arterial oxygen gradient (EPOC); SD, standard deviation; CI, confidence interval; LOA, limits of agreement. ^a^ mPaO_2_ obtained from the EPOC was corrected using the following equation: PaO_2_ corrected = 9.45 + mPaO_2_ × 0.90^20^.

**Table 3:** Multivariable linear analysis of factors associated with the discrepancy between cPaO_2_ and mPaO_2_.

Dependent Variable: cPaO_2_-mPaO_2_, mmHg	Full Model			Final Model		
	β-Coefficient	SE	*p* Value	β-Coefficient	SE	*p* Value
Intercept	−63.56	11.29	<0.001	−65.67	8.86	<0.001
Age, years	0.01	0.05	0.83	_	_	_
Male sex (vs. female)	−0.92	0.79	0.25	_	_	_
Body mass index, kg/m^2^	0.11	0.10	0.28	0.18	0.09	0.046
Baseline FEV_1_ (% predicted value) at 760 m	−2.66	3.78	0.48	_	_	_
SpO_2_, %	0.72	0.12	<0.001	0.70	0.10	<0.001
Time delay between end of AGM100 measurement and ABG puncture, sec	−0.001	0.002	0.54	_	_	_
PaCO_2_-PETCO_2_ difference, mmHg	0.53	0.16	0.002	0.47	0.13	<0.001

The final model was obtained after backward elimination of the non-significant variables (*p* > 0.05) from the full model. cPaO_2_, calculated arterial oxygen partial pressure (AGM100 device); mPaO_2_, measured arterial oxygen partial pressure (EPOC device); SpO_2_, oxygen saturation assessed by finger oximetry; FEV_1_, forced expiratory volume in the first second of expiration; PaCO_2_, partial pressure of arterial carbon dioxide; PETCO_2_, partial pressure of end-tidal carbon dioxide.

**Table 4:** Diagnostic accuracy of the AGM100 device to diagnose severe resting hypoxemia in COPD.

	Sensitivity	Specificity	PPV	NPV	LR+
PaO_2_ ≤ 55 mmHg	0.75	0.80	0.94	0.43	3.77
	(0.66 to 0.83)	(0.59 to 0.93)	(0.87 to 0.98)	(0.29 to 0.59)	(1.71 to 8.33)
PaO_2_ < 60 mmHg	0.86	0.80	0.99	0.18	4.29
	(0.78 to 0.91)	(0.28 to 0.99)	(0.95 to 1.00)	(0.05 to 0.40)	(0.74 to 24.77)

Estimates are presented with 95% confidence interval. PaO_2_ measurement obtained from aBGA (EPOC device) was considered as the reference test. COPD, chronic obstructive pulmonary disease; PPV, positive predictive value; NPV, negative predictive value; LR+, positive likelihood ratio; PaO_2_, arterial partial pressure of oxygen.

## Data Availability

Data are available upon reasonable request to the corresponding author.
